# Remote primary care during the COVID-19 pandemic for people experiencing homelessness: a qualitative study

**DOI:** 10.3399/BJGP.2021.0596

**Published:** 2022-04-05

**Authors:** Kelly Howells, Mat Amp, Martin Burrows, Jo Brown, Rachel Brennan, Joanne Dickinson, Shaun Jackson, Wan-Ley Yeung, Darren Ashcroft, Stephen Campbell, Thomas Blakeman, Caroline Sanders

**Affiliations:** NIHR Greater Manchester Patient Safety Translational Research Centre, University of Manchester, Manchester, and Division of Population Health, Health Services Research and Primary Care, Centre for Primary Care and Health Services Research, University of Manchester, Manchester.; Groundswell, London.; Inclusive Insight (previously director of research and campaigns, Groundswell, London).; Groundswell, London.; Groundswell, London.; Bolton NHS Foundation Trust, Bolton.; Urban Village Medical Practice, Manchester.; Salford Primary Care Together, Salford CCG, Salford.; NIHR Greater Manchester Patient Safety Translational Research Centre, Division of Pharmacy and Optometry, University of Manchester, Manchester.; NIHR Greater Manchester Patient Safety Translational Research Centre, University of Manchester, Manchester, and Division of Population Health, Health Services Research and Primary Care, Centre for Primary Care and Health Services Research, University of Manchester, Manchester.; Division of Population Health, Health Services Research and Primary Care, Centre for Primary Care and Health Services Research, University of Manchester, Manchester.; NIHR Greater Manchester Patient Safety Translational Research Centre, University of Manchester, Manchester, and Division of Population Health, Health Services Research and Primary Care, Centre for Primary Care and Health Services Research, University of Manchester, Manchester.

**Keywords:** COVID-19, healthcare disparities, homelessness, primary care, qualitative research, remote consultation

## Abstract

**Background:**

The COVID-19 pandemic has caused unprecedented disruption and change to the organisation of primary care, including for people experiencing homelessness who may not have access to a phone. Little is known about whether the recent changes required to deliver services to people experiencing homelessness will help to address or compound inequality in accessing care.

**Aim:**

To explore the experience and impact of organisational and technology changes in response to COVID-19 on access to health care for people experiencing homelessness.

**Design and setting:**

An action-led and participatory research methodology was employed in three case study sites made up of primary care services delivering care for people experiencing homelessness.

**Method:**

Individual semi-structured interviews were conducted with 21 people experiencing homelessness and 22 clinicians and support workers. Interviews were analysed using a framework approach.

**Results:**

The move to remote telephone consultations highlighted the difficulties experienced by participants in accessing health care. These barriers included problems at the practice level associated with remote triage as participants did not always have access to a phone or the means to pay for a phone call. This fostered increased reliance on support workers and clinicians working in the community to provide or facilitate a primary care appointment.

**Conclusion:**

The findings have emphasised the importance of addressing practical and technology barriers as well as supporting communication and choice for mode of consultation. The authors argue that consultations should not be remote ‘by default’ and instead take into consideration both the clinical and social factors underpinning health.

## INTRODUCTION

Homelessness is a persistent and growing problem in England and elsewhere.^1^ Shelter calculates that approximately 253 000 people are experiencing homelessness in England, with many thousands more at risk.^2^^,^^3^

People experiencing homelessness are heterogeneous, falling into multiple categories of homelessness including: roofless (rough sleepers or in emergency accommodation); houseless (those in homeless shelters); insecure (under threat of eviction); and inadequate (people living in temporary or unfit structures).^4^ The number of people falling into these categories is a moving picture, and the numbers in hotels and other temporary accommodation increased during COVID-19 lockdown periods. Recent evidence shows widening health inequalities for people experiencing homelessness^5^ with common experiences of both acute and ongoing complex mental, social, and physical care needs, and reduced life expectancy.^6^ Inequitable access to health care is identified as a key social determinant of health.^7^^,^^8^ Known as the ‘inverse care law’, those most in need of health care are often the least likely to receive it.^9^^–^11

The NHS Long Term Plan (2019) seeks to close the inequality gap and recommends prioritisation of underserved groups in primary care.^12^ Dedicated homeless and inclusion health services are available within mostly urban areas in the UK and they provide ‘drop-in’ and out-of-hours clinics and outreach health care.^13^^–^15 However, COVID-19 has disrupted this model of care with telephone or video consultations recommended to reduce infections.^16^ Pre-COVID-19, there were already calls to implement remote care via digital innovations to maximise staff capacity and access to primary care services.^12^ However, there were concerns about a ‘digital divide’^17^^,^^18^ and compounding inequalities for groups that are digitally excluded.^18^^,^^19^ Digital transformation to remote services including triage, as well as consultations, also raises questions about how to support people to access the right type of consultation (face-to-face, phone, or video) appropriate to their needs. This has implications regarding clinician–patient relationships^14^^,^^20^^–^22 such as relational continuity of care, which is viewed by the Royal College of General Practitioners (RCGP) as crucial in the diagnosis and treatment of conditions, especially among people with long-term or sensitive health conditions where stigma is often experienced.^23^^–^25

**Table:** How this fits in

Pre-COVID-19, there was mounting policy pressure to implement digital innovations to increase staff capacity and access to primary care services. There were concerns about a so-called ‘digital divide’ with certain population groups being left behind and excluded from potential benefits of technologies that could further exacerbate existing health inequalities. This qualitative study explores how the rapid change to remote care during the COVID-19 pandemic had an impact on access to, and safety of, care for people experiencing homelessness. It is argued that, although the remote model may facilitate more ‘timely’ access for some individuals, remote care should not be the default approach. The evidence highlights that the remote model may compromise a relationship-based approach, which is particularly important for building trust and continuity, and to mitigate against existing health inequalities.

Through data drawn from qualitative research methods, this article explores how people experiencing homelessness accessed primary care services during the COVID-19 pandemic. How clinicians and support workers (based in hostels or charities) provided and facilitated this care is also explored to gauge how the change to remote consultations has had an impact on access and service delivery within primary care.

## METHOD

An action-led and participatory qualitative research methodology was employed in three case study sites from September 2020 until the end of January 2021 (Box 1), made up of two commissioned primary care services and a community nurse inclusion health service. Three separate urban case study sites were chosen to compare organisational and local policy context. This included interviews with clinicians, stakeholders, and people experiencing homelessness. Action research and participatory methods refer to styles of research emphasising collaboration and democratic working between multiple partners to bring about change.^26^^,^^27^ It has been widely used for conducting community- or service-based research, including among people experiencing homelessness and service providers.^28^^–^31

**Box 1.** Description of case study sites

**Case study site**	**Service pre-COVID-19**	**Main changes during the COVID-19 pandemic**
CSS1 — primary care	Flexible drop-in appointments Outreach and hostel drop-ins Hospital in-reach service Case management of frequent attenders	No drop-in clinics except for wound care Telephone-led consultations Outreach was initially stopped and reintroduced in November 2020
CSS2 — primary care	GP service offering flexible appointments and drop-in service Outreach at day centres	No drop-in clinics Outreach was initially stopped but resumed in November 2020 Dedicated care coordinator to schedule appointments Video consultations trialled
CSS3 — NHS community nurse inclusion service	Nurse-led drop-in clinics Outreach in the community, hostels/guest houses/town centre walk-in clinic Weekly street kitchen	Outreach to hostels and town centre walk-in clinics initially stopped in the first national lockdown from end of March to mid-June 2020. Street kitchen stopped for the same period Nurse-led face-to-face clinics continued and outreach to guest houses continued on a needs basis

*CSS = case study site.*

Flexible semi-structured interviews were conducted with health professionals and other staff (such as care coordinators and managers) at each case study site. These staff also facilitated snowball sampling of additional stakeholders who worked closely with the case study sites (such as hostel workers, volunteer organisations). Owing to COVID-19, social distancing restrictions, all interviews were conducted remotely over the telephone and were audio-recorded. Interviews with clinicians and other stakeholders were conducted by the first author. To observe change in practice through the pandemic, one GP kept an audio diary to record their daily observations. The authors held team meetings with the primary care providers every 3 months to reflect on organisational changes and issues across the case study sites in ‘real time’, and to discuss emerging findings regarding barriers to accessing care. Notes were taken in these meetings but they were not audio-recorded.

The interviews with people experiencing homelessness were led by a researcher employed by Groundswell (a homeless charity) following a peer-research approach.^32^^–^34

Researchers with lived experience of homelessness receive research skills training (via Groundswell) to enable them to more easily engage with the target research population, who may be wary of participating in research. Their shared experience sought to promote a sense of trust and integrity, which encourages participation in research and may help people speak more openly about their experiences.^34^^–^36

As a result of social distancing measures, the authors engaged with local hostels, day centres, charities, and outreach healthcare workers in the case study sites to act as gatekeepers for recruitment. Sampling was pragmatic, aiming to maximise diverse participation but the final sample was dependent on responses to information disseminated via gatekeepers. Individuals were eligible to participate if they were currently homeless or had recent (during the pandemic) lived experience of homelessness. An information sheet and consent form were given to eligible participants and they contacted the researcher directly if interested. In some circumstances, the gatekeeper facilitated contact where participants did not have access to a phone.

Participants experiencing homelessness were asked to consent to a follow-up interview 3–6 months after the initial interview to reflect on their experiences of changes to accessing care during the pandemic. All interviews were audio-recorded using an encrypted digital recorder and were immediately downloaded to an encrypted university laptop. Audio files were then transferred to an approved university transcription provider as an encrypted file. Once transcribed, all audio-recordings were deleted and transcripts were pseudonymised before sharing with the wider team for data analysis.

Ethical approval was obtained from an NHS Research Ethics Committee (REC) committee. Interview schedules were developed from the research and policy literature, with some changes added following interviews and research meetings with primary care providers.

### Analysis

The audio-recorded interviews and audio diary were transcribed verbatim and analysed using a framework method, producing a matrix of summarised data and a structure to analyse and summarise data within key themes. It is particularly suited to enabling analysis effectively within teams of multiple researchers.^37^^,^^38^ The approach allows for analysis according to predefined themes and themes that emerge more inductively from the data.^37^

The initial analytical framework was completed in Microsoft Excel and structured by the main aspects of experience reflected in the literature on homelessness and access.^39^ The university research team worked closely with the research lead and researchers from Groundswell to do all stages of coding and thematic analysis. To increase rigour, the initial coding of the interview transcripts was conducted separately by the first author (University of Manchester) and the second author (Groundswell) to detect similarities and variations of interpretation across the sample.

A sample of transcripts was also read and coded by the last author (University of Manchester) and the third author (Groundswell) to develop the main and sub- themes. This formed the basis for further discussion and critical analysis for further iteration of the main themes within the wider team, including the primary care authors.

## RESULTS

### Sample characteristics

In total, 22 stakeholders, including GPs (*n* = 4), community and practice nurses (*n* = 7), other staff based in general practice (*n* = 2), and support workers based in hostels or working in outreach (*n* = 9) were interviewed across three case study sites in North West England. Twenty- one people experiencing homelessness were interviewed (Table 1), including six followup interviews to gauge any changes in accessing care over the course of the pandemic.

**Table 1.** Demographic characteristics of people experiencing homelessness who participated in the study (*n* = 21)

**Characteristic**	**Participants experiencing homelessness,** ***n***
**Sex**	
Male	18
Female	3

**Age, years**	
25–34	5
35–44	6
45–54	8
55–64	2

**Ethnicity**	
White British	18
Mixed race	2
Eastern European	1

**Qualifications**	
None	11
GCSE	8
Degree	1
Missing	1

**Number of chronic conditions**	
1	3
2	2
≥3	16

**Health conditions**	
Anxiety and depression	15
Alcohol- and drug-related issues	11

*CSE = General Certificate of Secondary Education.*

The findings from this study can be grouped into three themes:

- the impact of remote triage on accessing appointments;
- consequences of remote care for communication and therapeutic relationships; and
- facilitating access to care: collaborative working across health and social care boundaries.

### The impact of remote triage on accessing appointments

Experiences of remote care were mixed with some of the participants experiencing homelessness preferring the change as it offered them more convenient and timely access. This was particularly the case for participants who struggled to attend GP appointments physically because of anxiety or practical barriers and for participants who needed a medication review or a repeat prescription, as such requests were easily resolved over the phone.

However, several frustrations with the new COVID-19 lockdown-enforced appointment systems were reported. Participants missed the ability to ‘drop in’ at their GP practice or day centre for a same-day appointment.

All appointments needed to be made either online or by phone with patients advised to call early in the morning for a same-day appointment. This often resulted in long waiting times to get through, with some participants struggling to make appointments as they did not always have access to a phone or the funds to pay for the phone call:

*‘It is harder now because you’re just on hold all the time. So when you’ve got credit, it sort of runs out. If you’re on a contract it wouldn’t bother you.’*

(ID 14, Male participant experiencing homelessness, aged 35–44 years, white British, more than four chronic conditions)

*‘I found that really quite difficult with the phone calls and stuff like that. Like getting through the doctors. It was really hard work. Not all the time do I have* [access to] *a computer or like a phone with internet on it. The staff obviously here at the hostel are busy, so we can’t really use the phones or use the computer. You can’t even touch the computer because of the coronavirus pandemic that’s going on and social distancing. I just find it, like the whole … it’s really hard.’*

(ID 8, Female participant experiencing homelessness, aged 35–44 years, white British, four chronic conditions)

Once successfully through to the practice, patients then had to discuss their health concern with the GP receptionist to determine the urgency of their concern and the nature of the appointment required (triage). Participants were sometimes reluctant to discuss concerns with receptionists as they are not medically qualified, with some participants referring to them as strangers:

*‘Yeah, it’s an issue for me because I’ve got such a complicated medical history with having cancer and the permanent side effects from the treatments that I’ve got now, I don’t want to be explaining them to a stranger.’*

(ID 11, Male participant experiencing homelessness, aged 25–34 years, Eastern European, one long- term condition)

Once triage was completed, participants were not always given a specific appointment time, which sometimes resulted in missed or delayed consultations. Participants with immediate health issues expressed concern that a late call back would delay access to urgently needed medications or treatment. This resulted in some participants accessing care elsewhere, such as at a pharmacy or walk-in centre. This frustration was echoed by the community nurses interviewed, as they often had to support their patients to reorganise the telephone consultations they had missed as appointment times were not always specified:

*‘Like today I went into one of the hostels and a lady in there who I went to see, to provide wound care, she said that the GPs had phoned … they said they would phone her at nine o’clock and they didn’t ring her until four o’clock in the afternoon. So she missed that call because again, it wasn’t that they stuck to the agreed time.’*

(ID 6, Community nurse, 15 years’ experience)

### Consequences of remote care for communication and therapeutic relationships

Despite the possibility of delivering remote consultations by telephone or video, all the participants interviewed had only participated in a telephone consultation. At the beginning of the pandemic, video consultations were encouraged to bridge the gap between remote and face-toface care but the clinicians interviewed found this mode of consulting sometimes difficult and it was therefore rarely offered to patients experiencing homelessness:

*‘Speaking to patients who get a really poor reception and phones are cutting out. Some use Wi-Fi in the hostel and it’s not very good Wi-Fi and then there is a lag with the speech and the picture. It becomes pixelated and this is unhelpful. The technology has got to be good enough quality for us to do our work.’*

(GP 1, worked as a GP specialising in homelessness for 8 years, audio diary)

Two of the case study sites primarily preferred to use telephone consultations to provide care. At the practical level, both clinicians and participants noted that technical issues could impede communication during the consultation with poor phone signal a recurring problem.

The clinicians interviewed were concerned that telephone consultations could potentially compromise patient safety as opportunities for patients to discuss other health issues would not necessarily arise as non-verbal aspects of the consultations were described as ‘lost’, making it more difficult to provide a timely diagnosis and assess risk.

Body language was considered important in assessing the wellbeing of patients as often they struggled to discuss social and mental health issues with clinicians they did not always know or trust:

*‘It’s like clues that you pick up isn’t it from people’s non-verbal communication as well, just the way they present, their eye contact. Whether somebody’s depressed, withdrawn. A lot of these, it’s not just that … you can’t gather that over a telephone.’*

(ID 6, Community nurse, 15 years’ experience)

This view was shared by some participants who found it difficult talking to their GP over the phone, with the sensory aspects of the consultation implicitly linked to safety. This was reflected particularly in concerns highlighted about remote clinical assessment as a basis for diagnosis and management:

*‘Seeing a doctor face-to-face is a lot more personal, and then you can show the doctor where you’re hurting. I’m finding it quite difficult to understand how doctors can make diagnosis over a phone when they’re not seeing it.’*

(ID 11, Male participant experiencing homelessness, aged 25–34 years, Eastern European, one long-term condition)

The dynamics of the doctor–patient relationship were described as ‘transactional’ by some clinicians as consultations were primarily symptom focused. However, a pre-existing relationship between doctor and patient was considered to mitigate against the focus on diagnosis:

*‘If you have the right rapport with a patient I think this kind of consultation can still work but I guess I question new patients because it’s difficult to build that early rapport without being there in the same room as someone and there’s much more of a transactional exchange … Pre-existing relationships can make the consultation easier and less ‘transactional.’*

(GP 1, worked as a GP specialising in homelessness for 8 years, audio diary)

This dynamic was also reflected by some participants experiencing homelessness who struggled to verbalise their concerns, particularly their social situation, over the phone. One participant felt they received less ‘empathy’ over the phone, perhaps because of the more transactional nature of telephone consultations:

*‘If it was face-to-face so much easier because you can explain your situation to gain either empathy or … I don’t want sympathy but somewhere in the middle of these, do you know what I mean? But I’ll tell you what you get, you do get something in the middle because you get apathy.’*

(ID 3, Male participant experiencing homelessness, aged 45–54 years, white British, three chronic conditions)

Although some participants felt nervous and distrustful of remote care, others found the anonymity empowering as it avoided perceived judgement and therefore reduced anxiety:

*‘Because of the anxiety as well, I don’t really like associating with people, so it makes it* [having a consultation over the phone] *a bit easier for me.’*

(ID 10, Female participant experiencing homelessness, aged 45–54 years, white British, one chronic condition)

### Facilitating access to care: collaborative working across health and social care boundaries

The three case study sites often worked in collaboration with community and social care sectors pre-pandemic. The pandemic highlighted the importance of this collaborative approach with hostel and charity outreach workers being key facilitators of care. This included making appointments/medical queries on behalf of people experiencing homelessness, supplying pre-paid phones and digital devices to hostels (such as iPads), to enable easier and timely access, engaging with people to raise awareness of organisational changes, facilitating GP registration, and discussing specific patients as part of a multidisciplinary team meeting within the primary care network.

Not all the participants interviewed were registered with an enhanced general practice or had access to support via a hostel or outreach service. In one case study site (site 3), care continued to be provided face-to-face by the community nurse team during the first and subsequent lockdowns via both a walk-in clinic and outreach care. However, as the only face-toface provider of care in the local area, their roles evolved to become not only providers of care but also ‘mediators’. This involved making appointments on behalf of patients and facilitating appointments by allowing their patients to use their smartphones. The nurses interviewed often went above and beyond their expected role and working hours to secure appointments for their patients. As one nurse described, they became the ‘eyes and ears’ of the GP:

*‘I think the main thing is, is not being able to access GPs for patients, you know, most patients don’t have telephones to make telephone calls. So they would come to our clinic, and if they needed a telephone consultation with the GPs, they would ask us if we would be able to do that for them. And then you try ringing the GP up, and unfortunately you have to ring at a certain time, which is eight o’clock in the morning. Now I don’t work at eight o’clock, but I know in the past I have rung to make appointments for patients at eight o’clock in the morning, you know, which can be … you know, I’m not in work, I’ve had to go out of my house to do this telephone call.’*

(ID 10, Community nurse, 5 years’ experience)

The community nurse team was particularly helpful for participants who were not currently registered with a GP and were either unaware of their right to register or they lacked the confidence to challenge general practices that refused registration requests. The community nurses would advocate for their patients and ensure that they were not illegitimately turned away from practices. Over the course of the pandemic, this prompted a new partnership between the community nurses and local GPs who worked together to manage the complex needs of people experiencing homelessness:

*‘GPs have had to learn a new way of working. It’s not easy for them because they’ve had to learn new telephone skills and trying to do consultations over the phone without actually visually looking at a patient. We have had issues where we’ve actually gone to see a patient because they couldn’t get a GP appointment. We’ve basically been the GPs eyes, to sort of say, look X, Y, and Z is happening here … Yeah, it’s very difficult. I mean, we do feel that we’ve plugged the gap for a lot of services.’*

(ID 6, Community nurse, 15 years’ experience)

However, the community nurse team and support workers interviewed found it more difficult to facilitate for people registered with a mainstream primary care service as they were not always aware of or had received inclusion healthcare training:

*‘You know, our patients trust us, they respect us, we don’t get any grief from anybody. Our patients unfortunately get grief from other health professionals, which isn’t fair. It’s judgemental, but you know unless you work with people who are dependent on drug or alcohol or both, plus have mental health issues, unless you work in that field all the time, it is difficult not to judge people.’*

(ID 15, Practice nurse, advanced nurse practitioner)

For this reason, there was concern from the support workers and clinicians as to ‘who was being missed’ as it was acknowledged that not all people experiencing homelessness would have access to a support worker or outreach team to facilitate the healthcare system for them. For this reason, the support workers interviewed often encouraged their clients to register with an enhanced GP service if possible as these services were more aware of the barriers faced by people experiencing homelessness during the pandemic:

*‘I am aware that there are people now who will need an individual to help them access health services, an individual like myself who can liaise with somebody. In a way that introduces an extra barrier if you don’t have access to IT* [information technology] *. Whereas if there was a drop-in service that they could access at times that they chose themselves they may get to talk to somebody faster … I have quite close connections with homeless primary care services but if there’s another GP service there are a lot more barriers there, because they’re not used to dealing with a third party, they’ve not had any contact with me before. That’s why I would encourage somebody to register with a specialist homeless GP service unless they were very keen to remain at their current GP service.’*

(ID 62, Outreach worker, 20 years’ experience)

However, the GPs’ interviews highlighted that relying on support workers could eventually be problematic for anyone experiencing homelessness as they may lose touch with their support worker or no longer have access once they transition to living in permanent accommodation. For this reason, the GPs and nurses interviewed felt it was important to support and enable people to access healthcare services for themselves by offering a ‘flexible’ model of care that recognised that services could not be exclusively remote or digital:

*‘The system* [remote only] *how it currently stands will perpetuate inequality — there are positives but there are negatives but the people who will experience problems using it often don’t have a voice so it is sometimes assumed it is working.’*

(GP 2, 25 years qualified as a GP)

## DISCUSSION

### Summary

This study identified key access points and barriers to accessing, receiving, and delivering primary care, at a time of rapid transformation during the COVID-19 pandemic for people experiencing homelessness. Although some of the barriers existed pre-COVID-19, the move to remote telephone consultations brought sharply into focus the difficulties experienced by participants, including problems at the practice level associated with remote triage, as participants did not always have access to a smartphone to enable a video consultation or the means to pay for a phone call. This fostered increased reliance on support workers and clinicians working in the community to provide or facilitate a primary care appointment. At the interactional level, people experiencing homelessness and health staff found that face-to-face care that enabled eye contact and non-verbal communication was important for patient safety (including for those with complex needs and other vulnerable groups). Such direct interaction can be important for not only making a timely diagnosis and continuity of care, but also in enabling rapport and trust so that sensitive issues, such as mental and sexual health, were more likely to be discussed.

This study found that face-to-face interactions remained a fundamental aspect of delivering health care during the pandemic. This was delivered either by clinicians in general practice or the wider primary healthcare team, or facilitated by support workers and outreach teams. The findings highlight that the responsiveness and success of implementing a remote model for people experiencing homelessness relies heavily on flexible and collaborative working across health and community organisations. This involves nurses and support workers, having face-to-face contact and supporting access for remote consultations where needed.

### Strengths and limitations

This study endeavoured to capture a wide range of diverse experiences by engaging with local stakeholders and outreach workers to act as gatekeepers to recruit people experiencing homelessness. Although it is acknowledged that these findings will not be representative of all people experiencing homelessness, a key strength of this work is that it captured the patient and clinician experience. However, quantitative data may have provided more evidence on variation, such as mode of consultation and number of appointments.

Peer-led research is associated strongly with social change, inclusivity, and breaking down the traditional hierarchies between researchers and participants^34^ although some argue that peer-researchers can lack detachment from the research issue leading to bias in findings.^36^^,^^40^ However, a key strength is that all research interviews were reviewed by the core research team and feedback provided to help guide ongoing interviews.

### Comparison with existing literature

Much of the existing literature on the use of digital health innovations for people experiencing homelessness originates from the US.^41^ It has demonstrated that telephone consultations can improve access to care for patients and reduce workload for staff, and some research has pointed to enhanced benefits of video consultations, particularly when patients are anxious.^42^ Systematic reviews indicate that they can provide safe and high-quality care for ‘clinically appropriate’ patients, but most evidence has been based on people with stable chronic conditions.^17^^,^^43^

Greenhalgh *et al*^16^ suggest that, within the context of COVID-19, video and telephone consultations are unlikely to be appropriate replacements for seriously ill patients or for those who are not experienced at using digital technologies.^44^ Relationship-based care is a key strategic priority of the RCGP. A relationship-based care model is centred on a *‘high quality relationship between doctor and patient’*, in which continuity, compassion, and trust are deemed essential in improving the quality and safety of care.^22^ However, the RCGP has raised concerns that the relationship-based care model has been compromised by the transactional nature of remote telephone care during the pandemic.^22^^,^^45^ The research presented in the current study supports this view as the interviews with participants experiencing homelessness and clinicians alike highlight how implementing the relationship-based model can be more challenging when consulting remotely, particularly if there is no prior relationship or the patient is vulnerable or dealing with sensitive and complex health issues. The authors argue that the relational aspects of care are important in enabling access to care and reducing health inequalities. This relationship-based approach extends to non-clinical primary care staff who often engage with people experiencing homelessness at the first point of access. The current findings highlight the importance of having responsive and flexible organisational processes in place to enable a relationship-based approach at each stage of the patient journey.

### Implications for practice

Although a collaborative approach mitigated against some of the potential pitfalls of the remote model, there was recognition from the clinicians interviewed that not all people experiencing homelessness, particularly the ‘hidden’ homeless and people at severe risk of homelessness, would necessarily have someone to facilitate an appointment for them. For this reason, case study sites 1 and 2 adapted their organisational processes to accommodate the complex needs of people experiencing homelessness. There was recognition that long-term organisational changes were required to enable access, such as the re-introduction of ‘flexibility’ that had not been possible at the beginning of the pandemic. In the case study sites this included adapting appointment protocols so that people experiencing homelessness could bypass triage and be offered a face-to-face appointment if they appeared distressed or unable to articulate their health problem. Equally, the authors acknowledge that decisions regarding the mode of the consultation are complex; in some cases a telephone consultation may be most suitable for offering timely access to care. It is recognised that there are limits of workforce capacity in providing face-toface care for all patients and that certain trade-offs are inherent with this approach, particularly for the fostering of trusting relationships, reflected in the reticence of participants to discuss their health needs with a receptionist and their ambivalence regarding the safety of the care they were receiving. This study highlights that the development of support roles within primary care networks, such as care coordinators, are important not only in facilitating timely access to care but they also enable the embedding of relationship-based care into and across routine general practice.^46^

Although there are undoubtedly benefits to the remote model, the authors argue that remote care should not be the default approach. The vulnerability of the patient in addition to their clinical needs should be taken into consideration. The evidence highlights that a relationship-based approach is important for building trust and continuity, and to mitigate against existing inequalities. As suggested in recent related research, there should be more of a focus on collaborative working within the NHS (including secondary care and community pharmacy) and other services, such as charities, hostels, alcohol and drug services, and job centres that have specific knowledge and experience of working with people encountering homelessness and other vulnerable groups.^14^^,^^47^^,^^48^ Specific training on inclusion health for reception staff across primary care and not just for clinicians and others working in enhanced services would also be beneficial.
